# Supplementary material for: Serum Untargeted Metabolism Reveals the Mechanism of L. plantarum ZDY2013 in Alleviating Kidney Injury Induced by High-Salt Diet
Source: Nutrients. 2021 Nov 1;13(11):3920. doi: 10.3390/nu13113920 (PMC8620752; doi:10.3390/nu13113920)
Supplement: Supplementary file 1 [file nutrients-13-03920-s001.zip › nutrients-1405663-supplementary.pdf]

Supplementary Materials

Supplementary Table S1, Primer sequence information table for mouse cytokines and tight junction proteins.

Supplementary Figure S1 , Principal coordinate analysis (PCOA) was performed for different groups based on the Weighted Unifrac and Unweighted UnifracBeta distances. 2, Non-metric multi-dimensional Scaling (NMD) can reflect inter-group and intra-group differences of NSD and HSD through the distance between points. 3, QC sample total ion flow diagram (TIC). The response intensity and retention time of each chromatographic peak were basically overlapping, indicating that the variation caused by instrument error was small in the whole experiment. 4, QC sample correlation map and QC sample MCC. The instrumental analysis system is stable and the data can be used for subsequent analysis. 5, Some metabolites in serum and their metabolic pathways. 6. Clustering heat map of metabolites changing in different groups. Red indicates high concentration of metabolites and blue indicates low concentration of metabolites. 7, Spearman correlation coefficient matrix heat map between intestinal flora and serum metabolites. 8, The scatter plot reflects the correlation between a single significantly different metabolite and a significantly different genus. If there is a complete correlation between them, all data points fall on the fitting line. If the parts are related, the data points fall on both sides of the line. different colors represent different groups. All data are presented as mean ± SD. * P < 0.05; * * P < 0.01; * * * P < 0.001; * * * * P < 0.0001.

Supplementary Table S1

Primer sequence information	
β-actin-F	GCTCCTCCTGAGCGCAAGTA
β-actin-R	CAGCTCAGTAACAGTCCGCC
Ocln-F	GGACTGTCAACTCTTTCCGC
Ocln-R	CATTTATGATGAACAGCCCC
Cldn3-F	CTGTCTGTCCTCTTCCAGCC
Cldn3-R	CCACTACCAGCAGTCGATGA
Tjp1-F	GATCCCTGTAAGTCACCCAGA
Tjp1-R	CTCCCTGCTTGCACTCCTATC
FN-F	ATGAGAAGCCTGGATCCCCT
FN-R	GGAAGGGTAACCAGTTGGGG
IL-6-F	CTGCAAGAGACTTCCATCCAG
IL-6-R	AGTGGTATAGACAGGTCTGTTGG
IFN-γ-F	TGATTGCGGGGTTGTATCTG

IFN-γ-R	CTGTCTGGCCTGCTGTTAAA
SOD1-F	TAACTGAAGGCCAGCATGGGT
SOD1-R	GGTCTCCAACATGCCTCTCTTC
SOD2-F	CAGACCTGCCTTACGACTATGG
SOD2-R	GCTGAAGAGCGACCTGAGTTGT
TGF-β1-F	GTCACTGGAGTTGTACGGCA
TGF-β1-R	TCATGTCATGGATGGTGCCC
CCL4-F	GAAACAGCAGGAAGTGGGAG
CCL4-R	CATGAAGCTCTGCGTGTCTG
CCL5-F	CCACTTCTTCTCTGGGTTGG
CCL5-R	GTGCCCACGTCAAGGAGTAT

Supplementary Figure S1

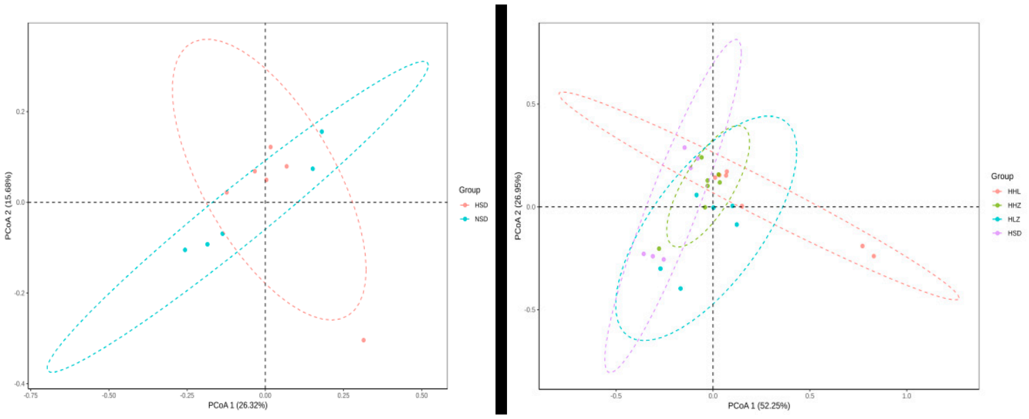

Supplementary Figure S2

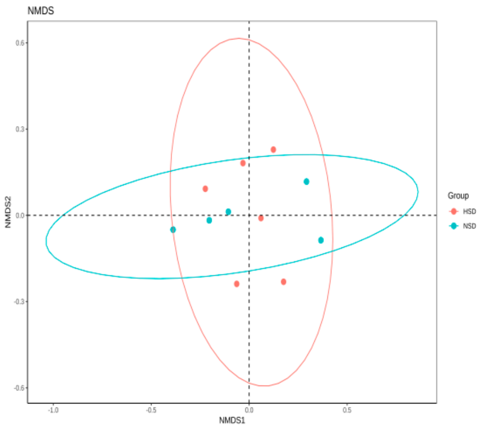

Supplementary Figure S3

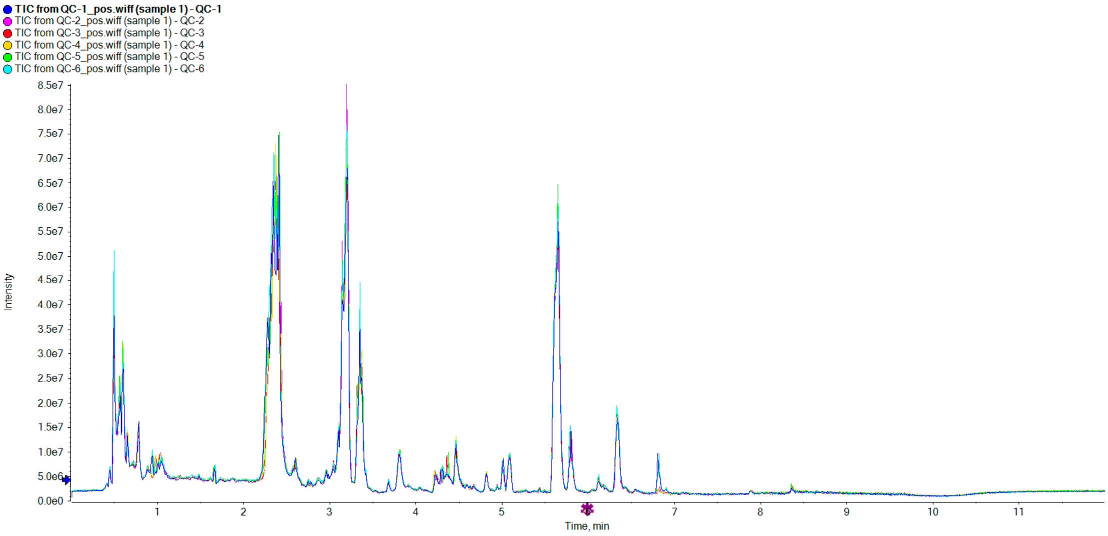

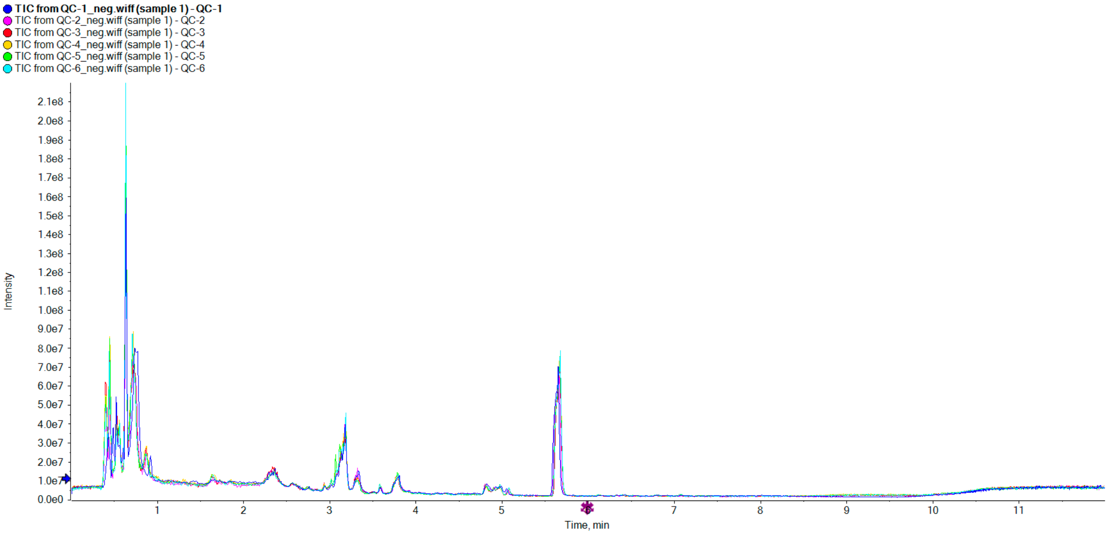

Supplementary Figure S4

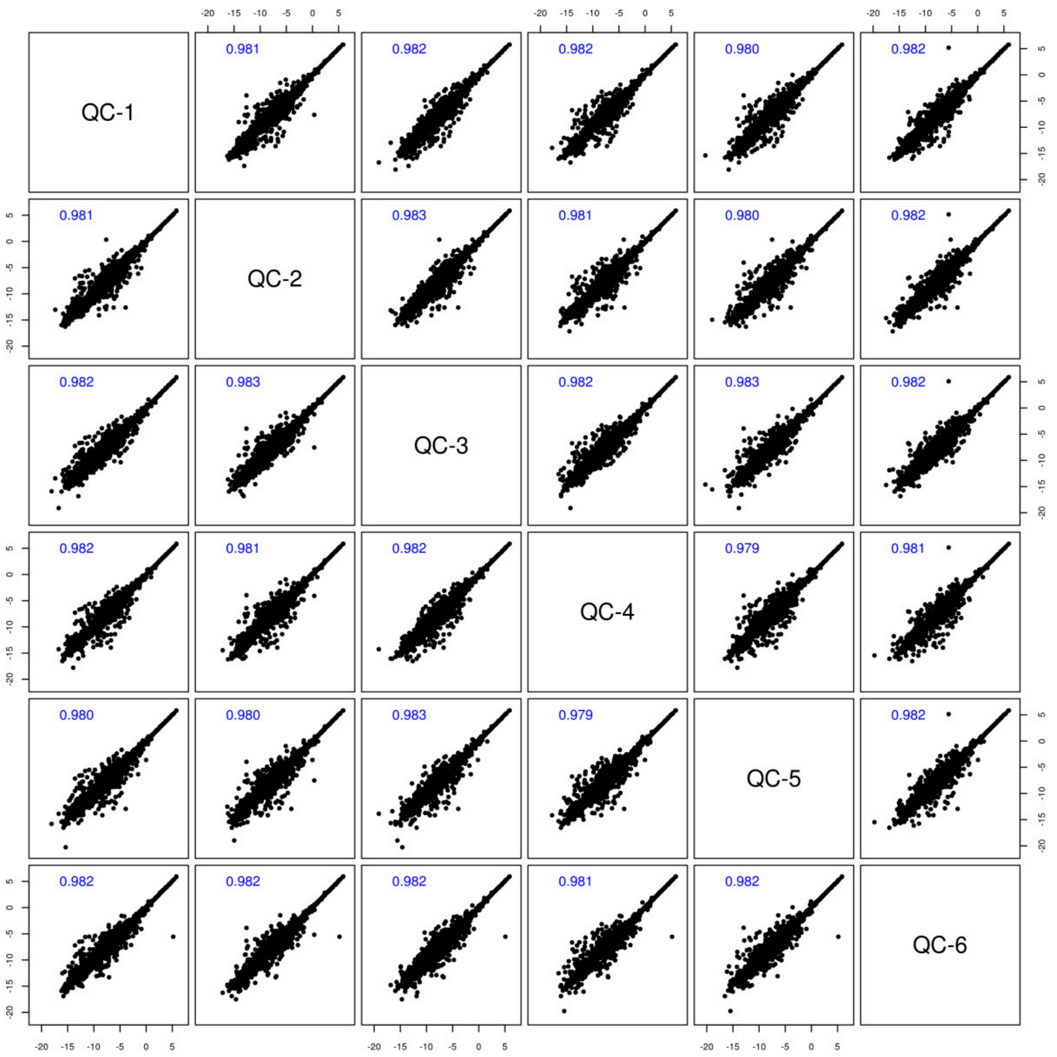
QC-1
QC-2
QC-3
QC-4
QC-5
QC-6
0.981
0.982
0.982
0.980
0.982
0.983
0.981
0.980
0.982
0.983
0.982
0.982
0.982
0.981
0.979
0.981
0.980
0.980
0.983
0.979
0.982
0.982
0.982
0.982
0.981
0.982
-20
-15
-10
-5
0
5

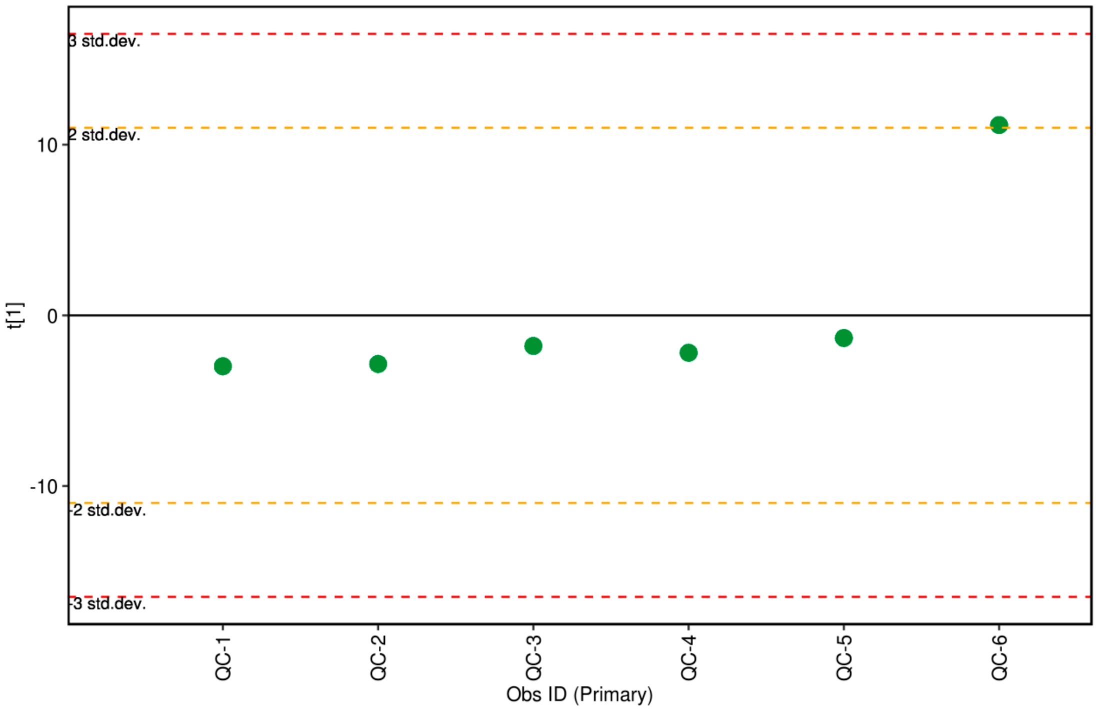
3 std.dev.
2 std.dev.
-2 std.dev.
-3 std.dev.
10
0
-10
t[1]
QC-1
QC-2
QC-3
QC-4
QC-5
QC-6
Obs ID (Primary)

Supplementary Figure S5

name	adduct	description	p value	m/z	rt(s)	DirectParent
M349T		Tetrahydrocortico	4.3830	349.2	179.	
179	(M-H)-	sterone	7E-09	3632	475	
M124T			2.1761	124.0	79.8	
80	(M-H)-	Taurine	1E-07	0641	29	Organosulfonic acids
M279T		alpha-Linolenic	1.2403	279.2	153.	Lineolic acids and
154	(M+H)+	acid	1E-06	3095	525	derivatives
M171T	(M+CH3	L-Pyroglutamic	3.4920	171.0	290.	Alpha amino acids and
290	CN+H)+	acid	7E-06	7604	167	derivatives
M298T		S-Methyl-5'-thioad	6.6240	298.0	94.4	5'-deoxy-5'-thionucleoside
94	(M+H)+	enosine	8E-06	9629	135	s
M363T		Hydroxyphenylla	2.4521	363.1	111.	
112	(2M-H)-	ctic acid	2E-05	1717	5955	Phenylpropanoic acids
M170T			2.6967	170.0	379.	
379	(M+H)+	3-Methylhistidine	3E-05	9164	298	Histidine and derivatives
M429T		alpha-Tocopherol	6.9251	429.3	129.	
129_2	(M-H)-	(Vitamin E)	9E-05	709	0005	Tocopherols
M182T			9.5124	182.0	40.7	
41_1	(M-H)-	4-Pyridoxic acid	1E-05	4487	94	Pyridinecarboxylic acids
M137T		1-Methylnicotina	0.0001	137.0	306.	
306_3	M+	mide	3863	6991	453	Nicotinamides
M163T			0.0002	163.0	200.	
200	(M-H)-	L-Fucose	90046	6017	0525	Hexoses
M220T	(M+CH3		0.0006	220.1	346.	
347	COO)-	L-Carnitine	03258	1779	8495	Carnitines
M309T	(M-H)-	2E-Eicosenoic acid	0.0006	309.2	39.5	

40			58658	7842	33	
M295T68	(M+Na)+	16-Hydroxypalmitic acid	0.000737318	295.22601	67.53	Long-chain fatty acids
M305T40_2	(M-H)-	Dihomo-gamma-Linolenic Acid	0.00077257	305.24693	39.533	
M223T200_2	(M+CH3COO)-	D-Quinovose	0.000874068	223.08133	200.04	
M511T45	(2M-H)-	Palmitic acid	0.001280396	511.47055	44.642	Long-chain fatty acids
M163T218	(M-H)-	L-Rhamnose	0.001524986	163.05989	217.749	Hexoses
M209T258	(M+H)+	L-Kynurenine	0.001583534	209.09104	257.6165	Alkyl-phenylketones
M201T25	(M+Na-2H)-	D-Mannose	0.001729695	201.03517	25.0535	Hexoses
M168T380	(M-H)-	1-Methylhistidine	0.001805227	168.07687	380.112	Histidine and derivatives
M269T211	(M+H)+	Inosine	0.00193314	269.08747	211.476	Purine nucleosides
M204T161	(M-H)-	Indolelactic acid	0.002291286	204.06546	161.002	Indolyl carboxylic acids and derivatives
M188T105	(M-H)-	3-Indolepropionic acid	0.005294514	188.07137	104.602	
M277T75	(M+H)+	Stearidonic Acid	0.006864044	277.21551	75.45	
M191T481	(M-H)-	Citrate	0.007751543	191.01857	480.5135	Tricarboxylic acids and derivatives
M118T347	(M+H)+	Guanidoacetic acid	0.008126027	118.06043	347.04	Alpha amino acids and derivatives

M104T364	M+	Choline	0.009564736	104.10604	363.7375	Cholines
M114T166_2	(M+H)+	Creatinine	0.012521519	114.0657	165.994	Alpha amino acids and derivatives
M176T133	(M+H)+	Indoleacetic acid	0.017092543	176.06991	132.804	Indole-3-acetic acid derivatives
M246T110	(M+NH4)+	2'-Deoxyuridine	0.019945043	246.10817	110.3565	Pyrimidine 2'-deoxyribonucleosides
M343T72	(M+CH3COO)-	Stearic acid	0.027799975	343.28284	72.029	Long-chain fatty acids
M149T132	(M-H)-	L-Arabinose	0.033174559	149.04471	131.566	Pentoses
M162T350_2	(M+H)+	L-Carnitine	0.034054086	162.11237	349.712	Carnitines
M190T99	(M+H)+	1H-Indole-3-propanoic acid	0.035424949	190.0853	98.867	
M157T180_2	(M-H)-	Allantoin	0.035457009	157.03616	180.434	Imidazoles
M139T150	(M+H)+	Nicotinamide N-oxide	0.037694317	139.0492	150.364	Nicotinamides
M684T491	(M+NH4)+	Stachyose	0.038260473	684.25403	491.483	Oligosaccharides
M451T161	(M+CH3COO)-	Chenodeoxycholate	0.038709683	451.30383	161.442	Dihydroxy bile acids, alcohols and derivatives
M258T248	(M+H)+	3-methylcytidine	0.045436603	258.10754	248.0145	
M347T495	(M-H)-	Inosine 5'-monophosphate (IMP)	0.04582318	347.03756	495.144	Purine ribonucleoside monophosphates

Supplementary Figure S6

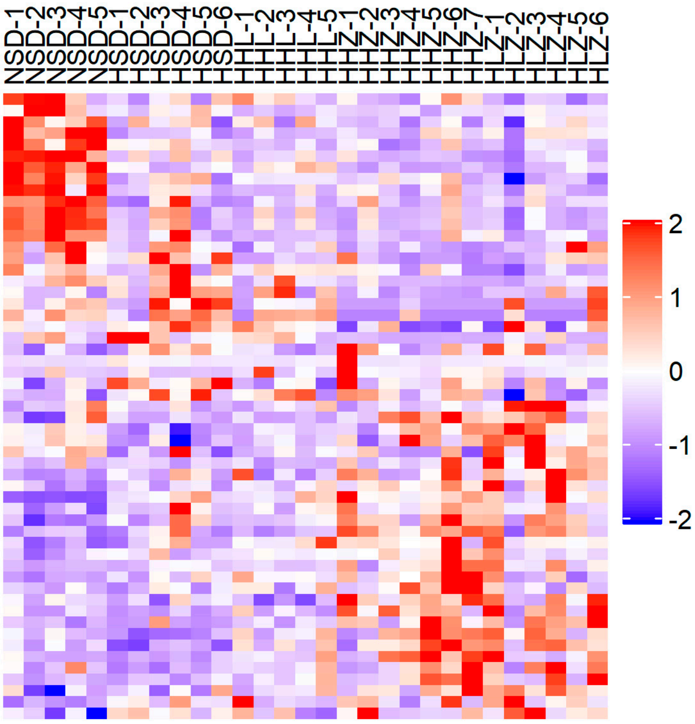

Supplementary Figure S7

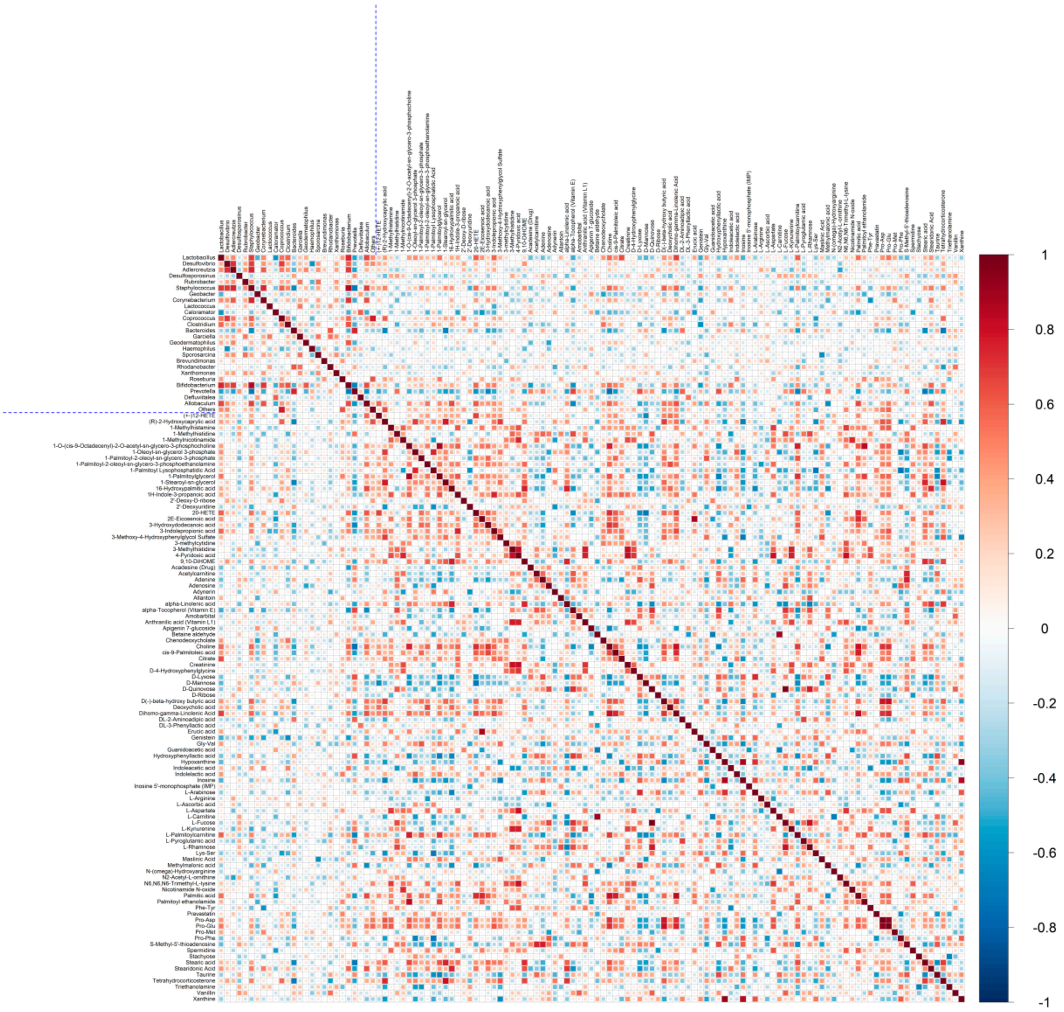

Supplementary Figure S8

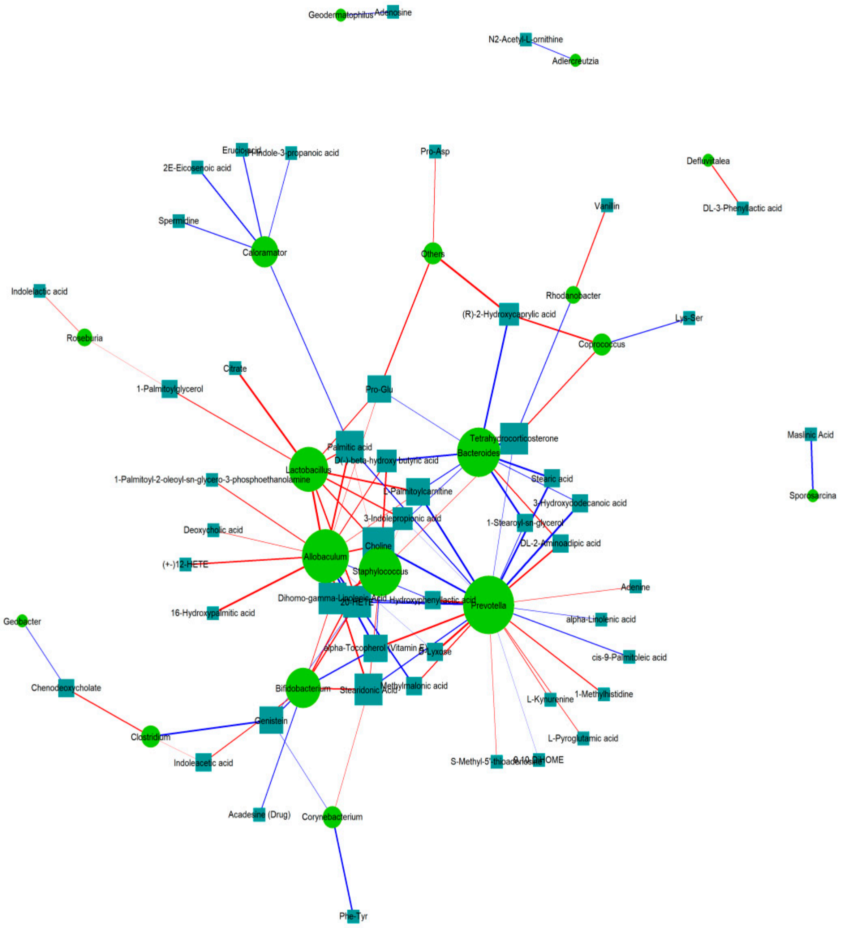

Geodermatophilus
Adenosine
N2-Acetyl-L-ornithine
Adlercreutzia
Erucic acid
1H-Indole-3-propanoic acid
2E-Eicosenoic acid
Pro-Asp
Defluviitalea
Vanillin
DL-3-Phenyllactic acid
Spermidine
Caloramator
Others
Indolelactic acid
Rhodanobacter
(R)-2-Hydroxycaprylic acid
Lys-Ser
Roseburia
Coprococcus
Citrate
1-Palmitoylglycerol
Pro-Glu
Maslinic Acid
Tetrahydrocorticosterone
Palmitic acid
DL-beta-hydroxybutyric acid
Lactobacillus
Bacteroides
Sporosarcina
1-Palmitoyl-2-oleoyl-sn-glycero-3-phosphoethanolamine
L-Palmitoylcarnitine
Stearic acid
3-Hydroxydodecanoic acid
3-Indolepropionic acid
1-Stearoyl-sn-glycerol
Deoxycholic acid
Choline
DL-2-Aminoadipic acid
Allobaculum
(+-)12-HETE
Staphylococcus
Adenine
Dihomo-gamma-Linolenic Acid
20-HETE
Hydroxyphenyllactic acid
Prevotella
16-Hydroxypalmitic acid
alpha-Linolenic acid
Geobacter
alpha-Tocopherol
Vitamin E
D-Lyxose
cis-9-Palmitoleic acid
Chenodeoxycholate
Bifidobacterium
Stearidonic Acid
Methylmalonic acid
L-Kynurenine
1-Methylhistidine
Genistein
Clostridium
L-Pyroglutamic acid
Indoleacetic acid
S-Methyl-5'-thioadenosine
9,10-DiHOME
Acadesine (Drug)
Corynebacterium
Phe-Tyr
